# Advanced dermatofibrosarcoma protuberans: an updated analysis of cases from an Indian sarcoma clinic

**DOI:** 10.2144/fsoa-2020-0198

**Published:** 2021-06-30

**Authors:** Azgar A Rasheed, Adarsh Barwad, Ekta Dhamija, Rakesh Garg, Rambha Pandey, Shamim A Shamim, Sreedharan T Arun Raj, Sameer Rastogi

**Affiliations:** 1 Department of Medical Oncology, BRA-IRCH, All India Institute of Medical Sciences, New Delhi, 110029, India; 2 Department of Pathology, All India Institute of Medical Sciences, New Delhi, 110029, India; 3 Department of Radiodiagnosis, BRA-IRCH, All India Institute of Medical Sciences, New Delhi, 110029, India; 4 Department of Onco-Anaesthesia & Palliative Medicine, BRA-IRCH, All India Institute of Medical Sciences, New Delhi, 110029, India; 5 Department of Radiation Oncology, BRA-IRCH, All India Institute of Medical Sciences, New Delhi, 110029, India; 6 Department of Nuclear Medicine, All India Institute of Medical Sciences, New Delhi, 110029, India

**Keywords:** advanced DFSP, dermatofibrosarcoma protuberans, fibrosarcomatous DFSP, imatinib, metastatic DFSP

## Abstract

**Aim:**

Dermatofibrosarcoma protuberans (DFSP) accounts for less than 2% of all soft-tissue sarcomas.

**Patients & methods:**

We retrospectively reviewed our database for patients with locally advanced or metastatic DFSP who had presented to our clinic between January 2016 and January 2020.

**Results:**

We identified a total of 14 patients, of whom ten had sarcomatous transformation. Eleven cases had metastatic disease and three were locally advanced. The initial partial response rate to first-line imatinib was 76.9% and the overall median progression-free survival on imatinib was 15 months.

**Conclusion:**

We had a high proportion of patients with sarcomatous transformation, in contrast to their relative rarity in the West. While most patients had initial good responses to imatinib, second-line therapies were not as effective.

## Lay abstract

Dermatofibrosarcoma protuberans is a rare sarcoma. Most patients have localized disease that can be cured by surgery alone. In those cases where either surgery is not possible or the disease is already metastatic, oral imatinib is the treatment of choice. In this article, we describe our experience with imatinib in such cases. Although most patients do well on imatinib, at least initially, many develop resistance to the drug. There are no established evidence-based therapies for patients who progress on imatinib, and as we describe in our paper, most of our patients did not do well on second-line medications.

Dermatofibrosarcoma protuberans (DFSP) is a rare tumor, accounting for less than 0.1% of all cancers and approximately 1% of all soft-tissue sarcomas. It is usually a low-grade malignancy, although 10–15% cases may be classifiable as intermediate grade due to a high-grade sarcomatous component (usually a fibrosarcoma [FS], DFSP-FS) [1]. It was first recognized as a distinct entity by Darier and Ferrand-Drake in 1924, and termed ‘dermatofibrosarkoma protuberans’ by Hoffman in 1925 [2,3]. It has an annual incidence of 0.8–4.5 per million and most commonly occurs in the fourth and fifth decades of life [4–6]. The most common primary site is the trunk, followed by the arms, the legs, and the head and neck region [6]. DFSP usually starts as an asymptomatic, slow-growing indurated plaque confined to the dermis, ranging in color from blue–red to violaceous or flesh colored, and eventually progresses to form a firm, protuberant nodular growth.

DFSP has a distinctive morphology, with a uniform population of monomorphic neoplastic spindle cells arranged in a predominantly storiform pattern over a background of fibrous stroma. There are no specific immunohistochemical markers for DFSP, but the tumor cells are usually positive for CD34 and vimentin on immunohistochemistry [7]. Chromosomal translocations involving 17q22 and 22q13 giving rise to a *COL1A1-PDGFB* fusion gene can be identified by fluorescence *in situ* hybridization (FISH) or real-time PCR in more than 90% of cases [8]. The *COL1A1* breakpoint can vary from exon 6 to exon 49, while the breakpoint in the *PDGFB* gene always occurs in the intron preceding exon 2 [8]. The specific position of the *COL1A1* breakpoint does not have any clinical or therapeutic implication [8,9]. The resultant fusion gene overcomes all negative controls on the translation and transcription of the *PDGFB* gene, causing an autocrine stimulation of DFSP tumor cell growth [10,11].

Localized tumors have a good prognosis. In a review of data from the SEER program for 1973–2002, the cause-specific 15-year survival rate was 99.7% (95% CI: 99.4–99.9%) [6]. The treatment for localized tumors is wide local excision (WLE) or Mohs micrographic surgery (MMS) with negative margins. There are no randomized trials comparing WLE versus MMS, and the choice between the two often comes down to availability, expertise, cost, the size of the tumor and its location. Achieving negative margins is often difficult due to the infiltration of tumor tissue into the subcutaneous planes, including fascia, muscle or bone, especially in recurrent cases. Higher age (>50 years), close (<1 mm) or positive surgical margins, DFSP-FS variant, high mitotic rate and increased cellularity are associated with decreased survival [1]. Local recurrence rates vary from 2 to 53% depending on the surgical techniques used for excision, but distant metastases are very rare (1–4%) [12–15]. In a series of 57 patients with DFSP, the mean time to the first recurrence after primary simple excision was 3.27 years for ordinary DFSP versus 1.87 years for DFSP-FS (p = 0.038) [16].

The US FDA approved imatinib for use in the management of unresectable, recurrent or metastatic DFSP in 2006 [17]. The largest prospective data for imatinib in advanced DFSP come from the pooled analysis of two Phase II trials conducted by the European Organisation for Research and Treatment of Cancer (EORTC) and the Southwest Oncology group (SWOG), with a total of 24 patients with locally advanced/metastatic DFSP given doses of imatinib ranging from 400 and 800 mg, respectively [18]. We had earlier published our experience with seven consecutive patients with metastatic/unresectable DFSP [19]. This updated analysis, after an interval of 3 years, includes seven additional patients with longer follow-up and also includes our experience with post-imatinib therapies in DFSP.

## Methods

We retrospectively reviewed cases from a prospectively collated database of patients who had presented to our medical oncology clinic between January 2016 and January 2020, with follow up till July 2020. We included all cases of metastatic or locally advanced DFSP. Cases where surgery was not feasible due to the site, extent of disease or risk of functional impairment, were considered to be locally advanced. All cases were discussed in a multidisciplinary clinic. The dose of imatinib was not standardized and depended on the physician’s discretion, clinical response and the tolerance of the patient.

Data including age, sex, primary site, histopathology, metastatic lesions, prior surgeries, prior radiotherapy, dose of imatinib, response rate, post-imatinib therapies and outcomes were extracted from hospital records. Data analysis was done using the IBM SPSS (Statistical Package for the Social Sciences) software for Windows, version 26.0.0 (IBM Corp., NY, USA). Baseline characteristics were assessed using descriptive statistics. Nominal data are presented as percentages and continuous data as median (range). Progression-free survival (PFS) was defined as time from the date of the first clinical visit at our center to the date of documented progressive disease or death from any cause. PFS was calculated using the Kaplan–Meier method. For univariate analysis, comparisons were made using the log-rank test.

## Results

### Patients

**Figure 1.**
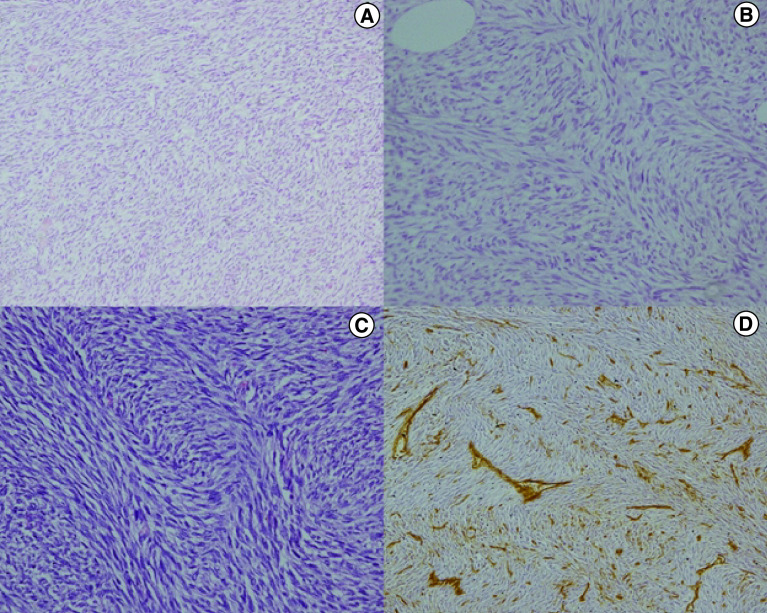
Histological picture from a case of dermatofibrosarcoma protuberans showing cells arranged in a storiform pattern. **(A)** High-power picture showing spindle cells that are showing mild nuclear atypia with elongated nuclei, finely dispersed chromatin and a moderate amount of eosinophilic cytoplasm. There is no mitosis and the overall mitotic activity is less than two per ten HPF (H&E ×100). **(B)** Histological picture from a case of fibrosarcomatous DFSP showing cells arranged in interlacing fascicles in a herringbone pattern. The individual tumor cells exhibit moderate nuclear atypia, a high nucleo-cytoplasmic ratio, hyperchromatic nuclei and scanty cytoplasm. Mitotic activity of around 8–10/10 HPF was noted (H&E ×200). **(C)** Immunostaining for CD34 showing diffuse loss of its expression in the spindle cell population in a case of fibrosarcomatous DFSP (H&E ×200). DFSP: Dermatofibrosarcoma protuberans; H&E: Hematoxylin and eosin staining; HPF: High-power field.

We identified a total of 14 patients who fit the inclusion criteria, with a median age of 39 years (range: 19–60 years). All cases were diagnosed based on histopathology and immunohistochemistry alone, by experts at a tertiary-level hospital (Figure 1). Molecular confirmation of the diagnosis was precluded by the unavailability of the tests in India. Patient characteristics are detailed in Table 1. Most of the patients (78.6%) were males. Ten of the 14 cases (71.4%) had sarcomatous transformation. The most common primary site was the trunk (12/14, 85.7%). One patient had a tumor involving the forehead and the adjoining medial orbital wall, and one had a tumor on the nape of the neck. Eleven cases had metastatic disease and the remaining three were locally advanced/unresectable. Among the metastatic cases, the median number of metastatic sites was 2 (range: 1–6), implying a relatively high burden of disease. The lungs were the most common site of metastases, being involved in nine of the 11 metastatic cases (81.8%). In the two cases with isolated lung metastases, the metastatic lesions were confirmed by biopsy. In the remaining patients, the timing of development of the lung lesions/their synchronicity with the appearance of lesions at other metastatic sites, and their response to imatinib was consistent with a clinical diagnosis of metastatic DFSP. Bone metastases were seen in four patients (36.3%) and soft-tissue metastases in three (27.3%). The median time from baseline diagnosis to development of metastasis was 45 months (range: 17–204 months). Our patients had undergone a median of three surgeries before being registered at our center (range: 0–5 surgeries) and half the cases (50%) had received radiotherapy previously.

**Table 1.** Clinicopathological characteristics of the study population and response to therapy.

Patient no.	Age (years)	Sex	Variant	*De novo* case or recurrence	Primary site	Stage	Sites of metastases	Prior surgeries (n)	Had previously received RT	Median dose of imatinib	Best response to imatinib	Progression on imatinib	PFS with imatinib (months)
1	33	M	DFSP	Recurrence	Forehead	Locally advanced	None	3	No	800	PR	No	38
2	54	M	DFSP-FS	Recurrence	Shoulder	Metastatic	Soft tissue	4	Yes	400	PR	Yes	7
3	48	M	DFSP-FS	Recurrence	Trunk	Metastatic	Lungs, stomach, ileum, pararenal lesions, bone	2	Yes	400	PD	Yes	1
4	35	M	DFSP-FS	Recurrence	Trunk	Metastatic	Lungs, kidney, liver, pancreas	1	Yes	600	PR	Yes	15
5	36	M	DFSP-FS	Recurrence	Trunk	Metastatic	Lungs, paraspinal soft tissue	4	No	800	PR	No	53
6	19	M	DFSP-FS	Recurrence	Trunk	Metastatic	Lungs, chest wall, axillary soft tissue	4	Yes	800	SD	Yes	17
7	35	F	DFSP-FS	Recurrence	Neck	Metastatic	Abdomen, lower limb, scalp, breast, lung, bones	1	No	600	PR	Yes	11
8	30	F	DFSP	Recurrence	Trunk	Locally advanced	None	4	No	800	PR	No	30
9	35	M	DFSP	*De novo*	Trunk	Locally advanced	None	0	No	400	PR	No	26
10	42	F	DFSP-FS	Recurrence	Trunk	Metastatic	Thigh	3	Yes	400	PR	Yes	4
11	50	M	DFSP-FS	Recurrence	Trunk	Metastatic	Lungs, bones	4	Yes	400	PD	Yes	3
12	49	M	DFSP-FS	Recurrence	Trunk	Metastatic	Lungs	5	No	400	PR	No	7
13	48	M	DFSP-FS	Recurrence	Trunk	Metastatic	Lungs, bones	2	Yes	400	PR	Yes	5
14	60	M	DFSP	Recurrence	Trunk	Metastatic	Lungs	3	No	–	–	–	–

DFSP: Dermatofibrosarcoma protuberans; DFSP-FS: Dermatofibrosarcoma protuberans with fibrosarcomatous transformation; PD: Progressive disease; PFS: Progression-free survival; PR: Partial response; RT: Radiotherapy; SD: Stable disease.

### Treatment outcomes

Three patients in our series had locally advanced disease and had excellent responses to imatinib. Patient 1 had presented to us with soft-tissue swellings over his forehead and the medial wall of the right orbit. He was started on imatinib at a dose of 400 mg once daily (OD) and achieved maximal response at 3 months of therapy with near complete resolution of the disease. A further increase in dose to 800 mg OD did not add to the response. The response achieved in this case with imatinib alone made surgery unnecessary.

Patient 8 had presented with an inoperable, recurrent lesion over her lower back, with a size of 15 × 15 cm. She was started on imatinib 400 mg OD, with which she had partial response after four months of therapy. The dose was then increased to 800 mg OD and after eight months of therapy, she was able to undergo WLE with split-skin grafting (SSG), followed by postoperative RT (PORT) at a dose of 60 Gy in 30 fractions over six weeks. She remains disease free 22 months after the surgery and is on regular follow-up. Patient 9 presented with a seven-year history of a pigmented, violaceous, plaque-like lesion over the left anterior chest wall that had gradually progressed to a size of 7 × 8 cm. He was given imatinib 400 mg OD for four months, with which he had partial response. He then underwent WLE with negative margins, followed by SSG and PORT. He has been on observation for 22 months since without disease recurrence.

Among the metastatic cases, one patient with a history of multiple recurrences, presented with two pulmonary metastases and no disease at the primary site. He successfully underwent metastatectomy with R0 resection and remains disease free 9 months later without any adjuvant therapy. Among the remaining ten metastatic cases, seven patients initially had at least a partial response (PR) with imatinib, one had stable disease (SD) and two experienced disease progression (PD) while on imatinib. Thus, the initial response rate to first-line imatinib was 76.9% (10/13) and overall disease control was achieved with imatinib in 84.6% (11/13). The median time from baseline diagnosis to the development of metastatic disease was 45 months (range: 17–204 months).

**Figure 2.**
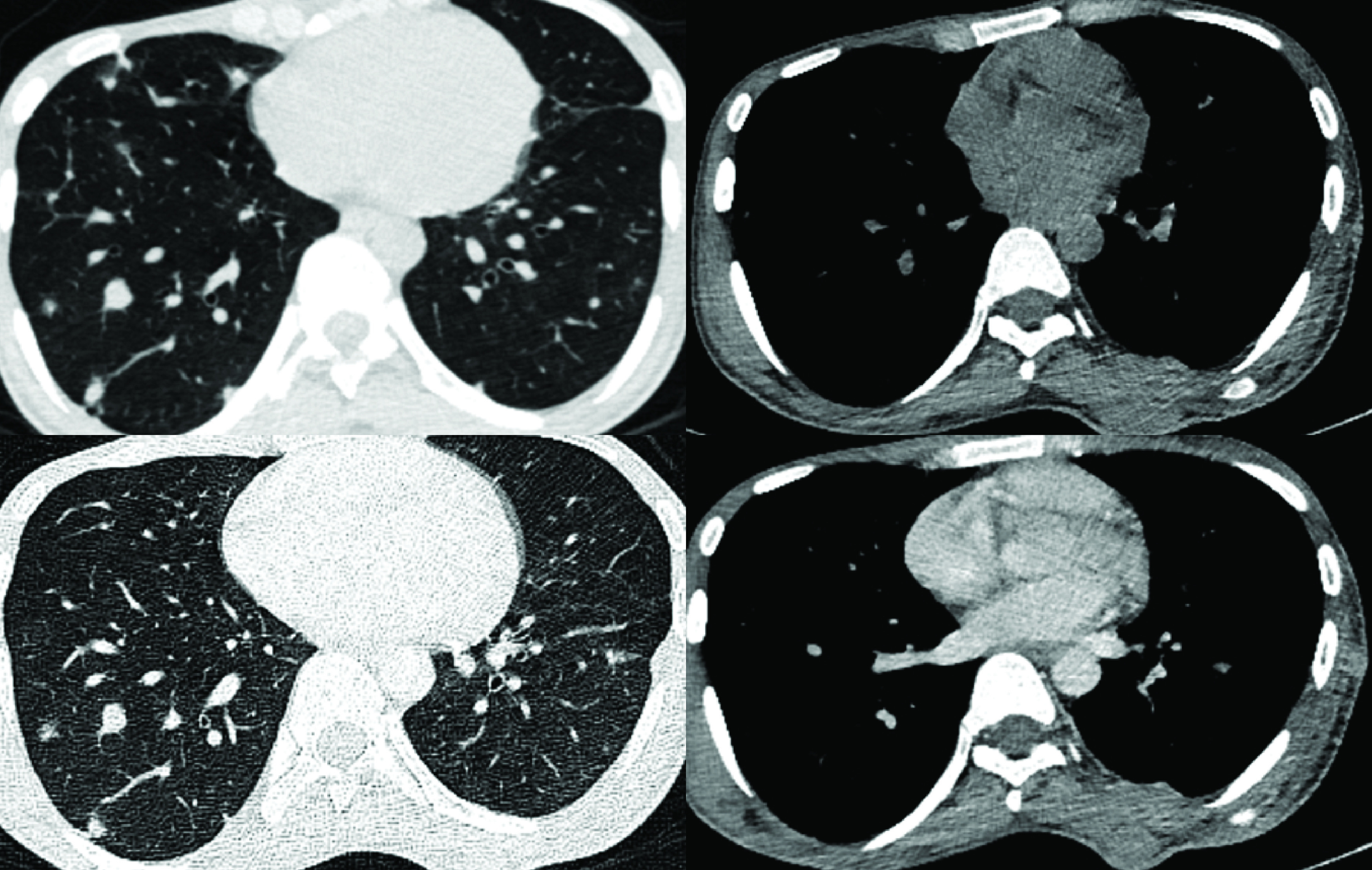
Contrast-enhanced computerized tomography images of patient 6, with dermatofibrosarcoma protuberans with fibrosarcomatous transformation lesions over the chest wall, in the axillary soft-tissue and in the lungs. After progressing on imatinib (top panel: June 2017), his disease has remained stable on pazopanib for 42 months (bottom panel: June 2020).

**Figure 3.**
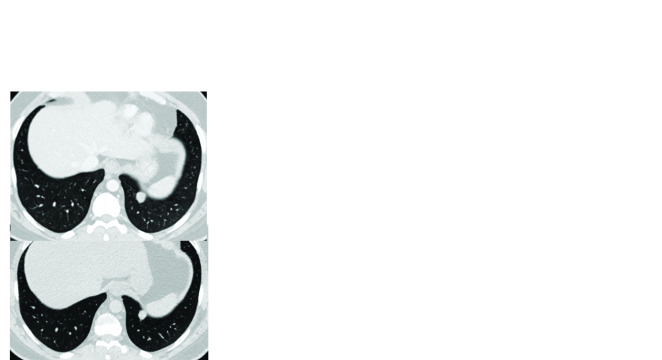
Contrast-enhanced computerized tomography images of patient 5, with a paraspinal mass and lung metastases, who continues to be progression free on imatinib at 53 months of therapy. Top image from July 2016 and bottom image from December 2019.

Patient 6 had developed metastatic disease within 3 years of initial presentation, and had received three cycles of ifosfamide-etoposide (IE) and vincristine-adriamycin-cyclophosphamide (VAC) at another center. He then had disease recurrence and lung metastases within two years, and underwent WLE followed by PORT (60 Gy in 30 fractions over 6 weeks), followed by pazopanib 400 mg OD, to which his best response was SD. The disease progressed after three years on pazopanib, at which time he presented to us. He was then started on imatinib 400 mg OD and had partial response. His disease progressed after 17 months on imatinib (Figure 2), at which point he was put back on pazopanib. His disease has now been stable on pazopanib for the past 42 months. Patient 5 (Figure 3) remains progression-free on imatinib at 53 months of therapy.

**Figure 4.**
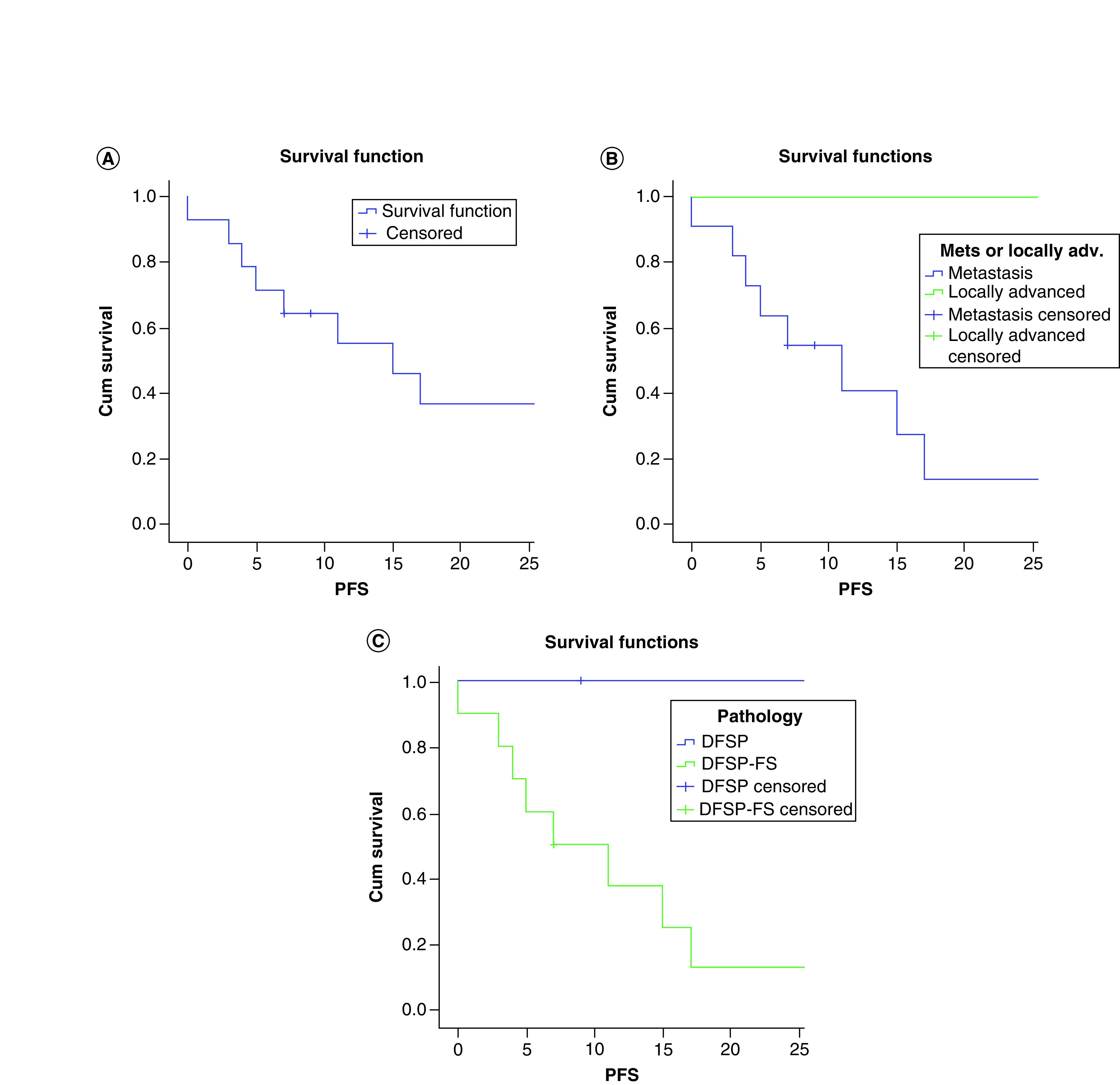
Kaplan–Meier curve for progression-free survival. **(A)** Overall, for all cases combined. **(B)** For patients with locally advanced versus metastatic disease. **(C)** For patients with DFSP versus DFSP-FS. DFSP: Dermatofibrosarcoma protuberans; DFSP-FS: Dermatofibrosarcoma protuberans with fibrosarcomatous transformation; PFS: Progression-free survival.

As shown in Figure 4A, the overall median PFS was 15 months (95% CI: 5.9–24.1 months) at a median follow-up of 17.5 months (range: 1–59). As shown in Table 2, of the eight cases who had progressive disease on imatinib, three were not fit for further therapy and three progressed within a month of second-line therapy: one each on dacarbazine, pazopanib and doxorubicin. Patient 2 received one cycle gemcitabine as third-line therapy, but the disease continued to progress, and he was only fit for best supportive care (BSC). Patient 4 progressed after two cycles of dacarbazine in the third line, and was re-exposed to imatinib at a dose of 800 mg. The disease remained stable on imatinib for eight months, after which the patient had PD and was advised BSC.

**Table 2.** Clinical response and survival of metastatic dermatofibrosarcoma protuberans patients on postimatinib therapies.

Patient no.	Initial response to imatinib	Median dose of imatinib	PFS on imatinib (months)	Second line	Response	Third line	Response
2	PR	400 mg	7	Pazopanib	PD after 1 month	Gemcitabine	PD after one cycle
3	PD	400 mg	1	Doxorubicin	PD after one cycle	BSC	–
4	PR	600 mg	15	Doxorubicin	SD after four cycles, PD after 6th cycle	Dacarbazine	PD after two cycles
6	SD	800 mg	17	Pazopanib	SD for 42 months, on follow up	–	–
7	PR	600 mg	11	Dacarbazine	PD after one cycle	BSC	–
10	PR	400 mg	4	BSC	–	–	–
11	PD	400 mg	3	BSC	–	–	–
13	PR	400 mg	5	BSC	–	–	–

BSC: Best supportive care; PD: Progressive disease; PFS: Progression-free survival; PR: Partial response; SD: Stable disease.

As shown in Figure 4B & C, on univariate analysis, cases with nonmetastatic disease (Chi-square: 4.67; p = 0.031) or DFSP pathology (Chi-square: 5.61; p = 0.018) were associated with a more favorable PFS. The median PFS on imatinib for the metastatic cases was 11 months (95% CI: 2.6–19.4 months) and the median PFS for the DFSP-FS cases was 7 months (95% CI: 1.0–15.3 months). There was no significant association between PFS and either imatinib dose (Chi-square: 3.145; p = 0.076) or gender (Chi-square: 0.097; p = 0.76).

## Discussion

The median age of patients in our study was 39 years (range: 19–60 years) compared with a median age of 47.4 years among the 24 patients in the pooled analysis of trials in advanced DFSP patients from the EORTC/SWOG groups [18]. The younger age at the onset of inoperable disease may be simply due to the younger demographic of the Indian population. In one series of 214 patients by David *et al.*, the mean age at which patients first noticed the lesions of DFSP was 29.6 years and the median diagnostic delay was 4 years [20]. The majority of the patients in our study were male (78.6%), compared with 58.3% in the EORTC/SWOG pooled analysis and 50% in the B5222 study [18,21].

FISH or PCR for COL1A1-PDGFB can be used for confirming a diagnosis of DFSP. Of the 50 patients with DFSP enrolled in the GENSARC study, molecular testing lead to a revised diagnosis in eight patients (16%) [22]. In the same study, three cases that had initially been diagnosed as being benign and two others with sarcomas with a different initial diagnosis were found to be DFSP on molecular analysis, with significant effects on disease management. Thus, molecular confirmation of the diagnosis in all our cases would have been ideal, but was not available. However, the good initial responses, we had in most of these cases attests to the validity of the diagnosis.

The overall incidence of fibrosarcomatous transformation in DFSP is reported to be 7–16% [1,23]. The incidence of fibrosarcomatous transformation in advanced/metastatic DFSP in the EORTC/SWOG and B2225 studies was 43–52%. In our series, ten of the 11 metastatic cases had DFSP-FS (90.9%). This may be explained by the high number of prior recurrences among the patients in our series. In a series of DFSP-FS cases by Mentzel *et al.*, the local recurrence rate was 58%, and 14.7% of cases developed metastatic disease [24]. The series of patients presented in this study include only those with advanced or metastatic disease. Metastatic disease is usually found in patients with a history of repeated surgeries. Among the DFSP-FS cases in our series, the median number of prior surgeries was three (range: 1–5). Seven of the 11 metastatic cases (63.6%) had also received radiotherapy for earlier recurrences. The high proportion of DFSP-FS among our metastatic cases has also been observed in other studies [25]. Fibrosarcomatous transformation is believed to contribute to an increased risk of recurrence and distant metastasis, and is considered by some to be a form of tumor progression [26]. The median PFS for the DFSP-FS cases was only 7 months (95% CI: 1.0–15.3 months), compared with the overall median PFS of 15 months.

Neoadjuvant imatinib is used at some centers to reduce tumor size and improve surgical outcomes, especially when the cosmetic results are important. It has had a response rate of 45–57% in Phase II trials [18,27]. Neoadjuvant doses of 400, 600 and 800 mg have been reported to have similar efficacy and tolerance profiles [18,27,28]. All three cases in our series with unresectable, advanced disease had good responses to imatinib 400 mg OD and higher doses did not bring increased benefits. The overall partial response rate to first-line imatinib in our series was 76.9% (10/13). The overall response rate to imatinib has been shown to be 46–90% in various studies, with shorter-lasting responses in fibrosarcomatous variants [18,21,29].

Imatinib has been shown to be effective in metastatic DFSP and is used as the first-line therapy. Among the ten metastatic cases exposed to imatinib in our series, seven had partial response and one had stable disease. The median PFS on imatinib for the metastatic cases in our series was 11 months (95% CI: 2.6–19.4 months). The overall median PFS was 15 months (95% CI: 5.9–24.1 months) at a median follow up of 17.5 months (range: 1–59 months), compared with a median time-to-progression of 1.7 years in the EOTRTC/SWOG series [18]. The lower PFS may be attributed to the high proportion of FS variants in our series.

The patient with limited metastases to the lungs, who is currently disease free after pulmonary metastatectomy, represents an interesting case study. However, the role of surgery versus continued imatinib therapy in limited metastatic disease is still under review. Perhaps, the absence of fibrosarcomatous transformation in this patient’s histology was also advantageous. Two of our patients had primary resistance to imatinib, and six cases had progressive disease after initial response, indicating secondary resistance. The mechanisms behind the development of this secondary resistance are still uncertain. In our series, six of 13 cases (46.2%) developed secondary resistance to imatinib after a median treatment duration of 6 months. In a series of 22 patients with locally advanced or metastatic DFSP, five of 22 patients (22.7%) developed secondary resistance to imatinib after a median treatment duration of 15 months [30]. Multiple case reports have documented the used of ifosfamide/adriamycin, dacarbazine, gemcitabine-docetaxel, pazopanib, sunitinib, sorafenib, nilotinib and methotrexate postprogression on imatinib, but none of these options have shown clear success. As such, DFSP is thought to be chemoresistant [31,32]. A multicentric Phase II trial of pazopanib in patients with unresectable or metastatic DFSP has reported an objective response rate of 30% [33], but further studies are required.

## Conclusion

The necessity of combined tumor boards (including medical, surgical and radiation oncologists) as well as multimodality treatment for the management of advanced DFSPs is clearly illustrated in our study. Our series had a high proportion of patients with sarcomatous transformation, in contrast to their relative rarity in western studies. Our patients had good outcomes with imatinib, and high initial response rate, both in locally advanced and metastatic disease. However, the results were dismal with subsequent therapies after progression on imatinib.

## Future perspective

Therapy for unresectable or metastatic carcinomas is currently mostly limited to imatinib alone. Although many drugs have been tried in the second line, there are only anecdotal reports of success with various agents. Prospective clinical trials, preferably with molecular testing, are required to identify better therapeutic options for these patients after progression on imatinib.

**Summary points**

- Dermatofibrosarcoma protuberans (DFSP) accounts for less than 0.1% of all cancers and approximately 1% of all soft-tissue sarcomas.
- Ten percent to 15% cases may be classifiable as intermediate grade due to a high-grade sarcomatous component (usually a fibrosarcoma [FS], DFSP-FS).
- The treatment for localized tumors is wide local excision or Mohs micrographic surgery with negative margins.
- Imatinib is the drug of choice for use in the management of unresectable, recurrent or metastatic DFSP.
- There is no significant difference in clinical outcomes with either 400 or 800 mg of imatinib.
- In our series of patients, 11 cases had metastatic disease and three were locally advanced.
- The median age was 39 years (range: 19–60 years), with males constituting 78.6% of patients.
- The most common primary site was the trunk (12/14, 85.7%).
- Among the metastatic cases, the median number of metastatic sites was 2 (range: 1–6), with the lungs being the most common site of metastases.
- The initial partial response rate to first-line imatinib was 76.9% (10/13) and the overall median progression-free survival on imatinib was 15 months (95% CI: 5.9–24.1 months) at a median follow-up of 17.5 months (range: 1–59).
- Ten cases (71.4%) had sarcomatous transformation. Fibrosarcomatous transformation is believed to contribute to an increased risk of recurrence and distant metastasis, and is considered by some to be a form of tumor progression.
- The median progression-free survival for the DFSP-FS cases was only 7 months (95% CI: 1.0–15.3 months), compared with the overall median progression-free survival of 15 months.
- There are no established therapies post progression on imatinib. Of the eight cases in our series who had progressive disease on imatinib, three were not fit for further therapy and three progressed within one month of starting second-line therapy: one each on dacarbazine, pazopanib and doxorubicin. One patient continues to have stable disease after 42 months of pazopanib therapy.
- Clinical trials are needed to establish second-line therapies in advanced/metastatic DFSP.
